# B serum proteome profiles revealed dysregulated proteins and mechanisms associated with insomnia patients: A preliminary study

**DOI:** 10.3389/fnint.2022.936955

**Published:** 2022-07-26

**Authors:** Tao Liu, Guanying Wang, Xingping Zhang, Xin Liu, Zhengting Liang, Xiaojuan Ren, Deqi Yan, Wenhui Zhang

**Affiliations:** ^1^Traditional Chinese Medicine Hospital Affiliated with Xinjiang Medical University, Ürümqi, China; ^2^Postdoctoral Workstation of Traditional Chinese Medicine Hospital of Xinjiang Uygur Autonomous Region, Ürümqi, China; ^3^Guizhou University of Traditional Chinese Medicine, Guiyang, China; ^4^College of Traditional Chinese Medicine, Xinjiang Medical University, Ürümqi, China; ^5^Xinjiang Urumqi Traditional Chinese Medicine Hospital, Ürümqi, China

**Keywords:** insomnia, protein expression, immuno-inflammation, PRM, sleep

## Abstract

**Background:**

Insomnia is a clinical problem of significant public health importance; however, the underlying pathogenesis of this disorder is not comprehensively understood.

**Methods:**

To identify potential treatment targets and unfold one of the gaps that were involved in insomnia pathological mechanisms, we employed a tandem mass tag-based (TMT) quantitative proteomics technology to detect differentially expressed proteins (DEPs) in serum from patients with insomnia and controls. DEPs were further analyzed by bioinformatics platforms. In addition, parallel reaction monitoring (PRM) was used to verify the TMT results.

**Results:**

Patients with insomnia had poorer sleep quality compared with healthy controls. A total of 106 DEPs were identified among patients with insomnia and controls. They were mainly enriched in immune and inflammation-related biological functions and signaling pathways. Using the protein–protein interaction network, we screened the 10 most connected proteins as key DEPs. We predicted that four key DEPs were subject to targeted regulation by natural compounds of herbs. Eight key DEPs were validated using PRM in an additional 15 patients with insomnia and 15 controls, and the results also supported the experimental findings.

**Conclusion:**

We identified aberrantly expressed proteins in insomnia that may be involved in the immune-inflammatory response. The 10 key DEPs screened may be potential targets for insomnia, especially FN1, EGF, HP, and IGF1. The results of this study will broaden our understanding of the pathological mechanisms of insomnia and provide more possibilities for pharmacotherapy.

## Introduction

Insomnia is a public health problem and one of the most frequent sleep disorders in patients with other neurological disorders (Sutton, 2021). This disorder is characterized by difficulties in guaranteeing sleep quality, initiating or maintaining sleep, and severe distress and impairment of daytime functioning (Bollu and Kaur, 2019). The prevalence of insomnia disorder is approximately 10–20%, of which approximately 50% have a chronic course (Buysse, 2013). Patients with insomnia are often accompanied by higher levels of depression, anxiety, physical pain and discomfort, cognitive impairment, and cardiovascular disease (Javaheri and Redline, 2017; Riemann et al., 2020). These disorders impose a personal and economic burden that is difficult to quantify (Wickwire et al., 2020). However, the underlying pathogenesis of this disorder is not completely understood. Therefore, it is necessary to study the mechanism of insomnia to better understand its pathophysiology and produce effective therapeutic agents.

It is important to note that both pharmacological and non-pharmacological treatments have their advantages and disadvantages, and neither treatment is suitable for patients with all types of insomnia (Hassinger et al., 2020). Molecular factors of sleep–wake regulation include sleep-promoting chemicals, such as norepinephrine and histamine, γ-aminobutyric acid (GABA), melatonin, etc. (Levenson et al., 2015). Abnormal regulation of these chemicals is one of the possible mechanisms of insomnia. Studies have shown that insomnia can alter inflammatory processes (Li et al., 2020). Inflammatory factors in turn may increase the response to insomnia and contribute to the etiology of physical and psychiatric disorders (Ferrie et al., 2013; Dolsen et al., 2019). Interventions to improve sleep and reduce inflammation may have a positive impact on health in the general population.

Lots of studies have reported that many proteins changed after insomnia, which were wisely related to the complex and dynamic pathophysiology (Xu et al., 2020; Wang et al., 2021). Quantitative proteomics has a popular profiling approach in protein biomarker discovery and protein alterations so far (Jha and Trivedi, 2019). Currently, with the advent of quantitative proteomic technologies using an isobaric labeling strategy, it becomes possible to quantify several proteins in a single experiment for the comparative study of global protein regulation across various biological samples, and this method has been widely applied to elucidate disease mechanisms (Kibinge et al., 2020; Kohlschmidt et al., 2021).

Identification of novel pathogenesis and new traditional Chinese medicines is made more possible by proteomics. Herein, we used TMT-based quantitative proteomics to explore the potential mechanisms and therapeutic targets for insomnia. We identified disordered proteins associated with insomnia by comparing the proteomes of patients with insomnia and controls. Based on the bioinformatic analysis, we aimed to reveal the novel processes involved in insomnia.

## Materials and methods

### Participants and selection criteria

All study protocols were approved by the Ethics Committee of the First Affiliated Hospital of Xinjiang Medical University, Urumqi, China (Grant No. 20120220-133). Written informed consent was signed by eligible participants (or their legal guardians). All participants with 40 primary insomnia complaints and 4 well-sleeping controls were collected from the First Affiliated Hospital of Xinjiang Medical University and Traditional Chinese Medicine Hospital Affiliated with Xinjiang Medical University. Patients who met the inclusion criteria were included in the study. The diagnosis was provided by the same doctor.

Patients were recruited to meet the following inclusion criteria: (i) Meeting the Diagnostic and Statistical Manual of Mental Disorders (fifth edition) criteria for insomnia disorder (American Psychiatric Association [APA], 2013); (ii) age ≥20 and ≤50 years. The exclusion criteria were as follows: (i) Use of psychotropic substances or not discontinued for more than 2 weeks; (ii) Insomnia caused by drinking alcohol and taking central stimulant drugs; (iii) A history of major neurological, psychiatric, or other medical disorders; and (iv) Pregnancy or lactation. Additionally, healthy controls were recruited from healthy volunteers.

Pittsburgh Sleep Quality Index (PSQI) was calculated for the patients. Polysomnography (PSG) data were used to assess standard sleep architecture variables. Healthy controls self-reported that they were not having any symptoms of insomnia.

### Blood sample collection and preparation

The subjects were fasted and watered for at least 12 h before drawing blood at 7–8 am. The blood samples were taken specifically for this study and coded to remain anonymous. A 3-ml blood serum sample was obtained from each enrolled subject. The blood samples were centrifuged at 2,000 × *g* for 15 min (4°C) and the serum was aliquoted. Then, the serum sample was divided into 0.5 ml aliquots and stored in the freezer (−80°C) until further analyses.

Serum samples from five patients with insomnia (G group) and five healthy controls (F group) randomly selected from all participants were analyzed by proteomics in the discovery phase. Furthermore, additionally, serum samples from 15 patients with insomnia and 15 healthy controls were randomly selected from all participants for parallel reaction monitoring (PRM) validation.

### Tandem mass tag-based quantitative proteomics

Pooled serum samples were generated by combining the same volume of individual serum samples in each group. The high-abundance proteins in the serum of each group were removed using the Pierce™ Top 12 Abundant Protein Depletion Spin Columns Kit (Thermo Fisher, Waltham, MA, United States). Finally, Protein concentration was done using a BCA kit (Sigma, St. Louis, MO, United States) according to the manufacturer’s instructions. For each sample, 50 μg of serum proteins was reduced using 5 mM dithiothreitol (Thermo Fisher, United States), and alkylated using 11 mM iodoacetamide (Sigma, United States). The proteins were then diluted with 50 mM Tris-HCl (pH 8.0) to reduce urea concentration and digested with trypsin. The resulting **peptide was desalted by Strata X C18 SPE column (Phenomenex)** and vacuum-dried. The peptide was reconstituted and processed according to the manufacturer’s protocol (Thermo Fisher, Waltham, MA, United States) for the TMT kit.

The mixed peptides were fractionated into fractions by high pH reverse-phase HPLC using Thermo Betasil C18 column (5 μm particles, 10 mm ID, 250 mm length). Then, the TMT-labeled tryptic peptides were reconstituted with 0.1% formic acid (solvent A) and loaded onto a homemade reversed-phase analytical column (15 cm length, 75 μm i.d.). The peptides were separated by UPLC and implanted into the NSI ion source for ionization, and then, analyzed by tandem mass spectrometry (MS/MS) in Q Exactive™ Plus (Thermo). The electrospray voltage was set to 2 kV. The scanning range of the primary-level MS was 350–1,800 m/z, and the scanning resolution is 70,000. The scanning range of the second-level MS was 100 m/z with a resolution of 17,500.

### Proteomics data analysis

The resulting MS/MS data were processed using MaxQuant (v.1.5.2.8). The retrieval parameters were set as: the database was human_swissprot_9606; enzyme mode was set to Trypsin/P; maximum missed cleavage number was 2; the mass tolerance for precursor ions was set as 20 ppm in the first search and 5 ppm in the main search; the mass tolerance for fragment ions was set as 0.02 Da; the signal threshold was set as 100,00 ions/s; maximum injection time set to 200 ms; false discovery rate (FDR) was adjusted to <1%. Protein quantification data with *p* < 0.05, ratio-fold change > 1.2 (upregulation) or <0.83 (downregulation) were considered as significant differentially expressed proteins (DEPs). Data in this study was submitted to the PRIDE database with an internal ID of PXD026610.

### Enrichment analysis

To annotate the potential functions of DEPs, the GO and KEGG pathway enrichment analysis was performed using the clusterProfiler R package (Yu et al., 2012). A *p* < 0.05 was considered statistically significant. Gene set variation analysis (GSVA) was performed using the GSVA R package to evaluate the activation of the KEGG pathway. In addition, the hallmark was obtained using a gene set enrichment analysis (GSEA) software.

### Protein–protein interaction network

All DEPs were searched against the STRING database version 11.0^1^ for protein–protein interaction (PPI) networks. The PPI network was visualized by the Gephi software. To identify the connectivity degree of proteins in the PPI network, the top ten proteins with the largest degree were considered as the key DEPs. The interaction network of key proteins was displayed with the Cytoscape software.

The Traditional Chinese Medicine Systems Pharmacology (TCMSP) database (Ru et al., 2014) was used to obtain natural compounds of herbal medicines. According to the criteria suggested by the TCMSP, we predicted natural compounds for key DEPs.

### Parallel reaction monitoring-mass spectrometry analysis

Targeted proteomics analysis, the PRM, was used to validate key DEPs screened by TMT analysis. PRM analysis defined the signature peptides of the target protein according to the TMT data. A unique peptide included for analysis in a minimum number of subjects was identified. More than two unique peptides per protein were used for quantification using fragment ion peak areas of peptides. The serum samples were prepared, reduced, alkylated, and digested with trypsin following the protocol for TMT analysis. The obtained peptide mixtures were separated and analyzed with nano-UPLC (EASY-nLC 1000) and MS/MS in Q Exactive™ Plus (Thermo). The resulting MS data were processed using the Skyline software (v.3.6). Peptide parameters are as follows: enzyme was set as Trypsin [KR/P]; Max missed cleavage was 0. The peptide length was set at 7–25; the alkylation of cysteine was set as a fixed modification. The parameters of transition were set as follows: precursor charges were set to 2, 3; ion charges were set as 1; ion types were set as b, y. The fragment ions were set from ion 3 to the last ion, and the ion match tolerance was set as 0.02 Da.

### Statistics

Statistical analysis was analyzed by the SPSS software (version 23.0). Results were compared by independent *t*-test or chi-square analyses. All data were expressed as mean ± SD, *p* < 0.05 was considered statistically significant.

## Results

### Baseline characteristics

**FIGURE 1:**
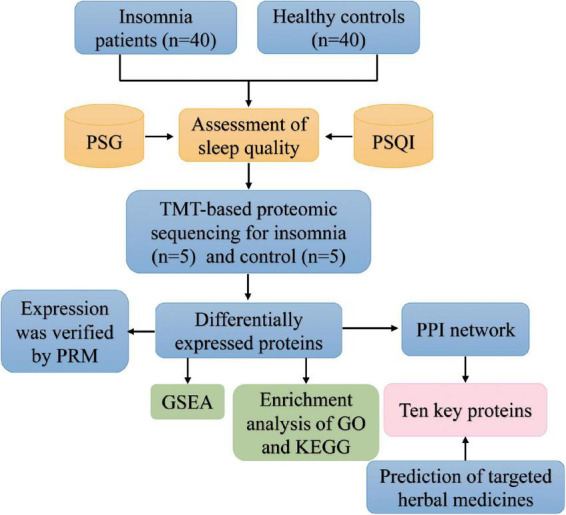
The flowchart of this study. GO, Gene Ontology; GSEA, gene set enrichment analysis; KEGG, Kyoto Encyclopedia of Genes and Genomes; PPI, protein–protein interaction; PRM, parallel reaction monitoring; PSG, polysomnography; PSQI, Pittsburgh Sleep Quality Index.

A flowchart of the study is shown in Figure 1. Participants in this study included primary insomnia complaints (*n* = 40) and well-sleeping controls (*n* = 40). The demographic and baseline data of registered subjects are shown in Table 1. Participants tended to be average-aged of 41.85 ± 5.27 in the insomnia group and average-aged of 41.06 ± 7.64 in the control group. Likewise, there were no significant differences between the insomnia and controls on sex and body mass index. In terms of sleep disorders, patients with insomnia had significantly worse sleep quality than healthy controls.

**TABLE 1:** Baseline characteristics of participants.

Characteristic	Insomnia group	Healthy control group	*t*/χ^2^	*P*
	(*N* = 40)	(*N* = 40)		
Age, y				
Mean (SD)	41.85 (5.27)	41.06 (7.64)	0.54	0.59
Median (range)	43.5 (31–50)	43 (25–50)		
Sex				
Male	13	17	0.85	0.36
Female	27	23		
BMI, mean (SD), kg/m^2^	24.3 (3.3)	23.64 (3.43)	0.88	0.38
PSG parameters, mean (SD)				
Time in bed, min	512.58 (65.12)	491.11 (33.51)	1.85	0.07
Total sleep time, min	388.68 (74.95)	449.88 (32.59)	4.74	<0.001
Sleep efficiency (%)	75.91 (11.47)	91.64 (3.62)	8.27	<0.001
Sleep-onset latency, min	34.38 (21.82)	8.05 (11.40)	6.76	<0.001
Awakening times	10.25 (5.08)	4.53 (2.74)	6.27	<0.001
Awake time, min	72.86 (54.70)	31.28 (16.94)	4.59	<0.001
REM sleep, min	68.2 (35.84)	63.71 (32.16)	0.59	0.56
REM%	17.2 (7.60)	14.13 (7.06)	1.87	0.06
NREM sleep, min	321.0 (60.86)	384.4 (41.46)	5.45	<0.001
NREM%	83.0 (7.47)	85.47 (7.02)	1.52	0.13
PSQI score, mean (SD)	14 (2.30)	–		

BMI, body mass index; NREM, non-rapid eye movement sleep; PSG, polysomnography; PSQI, Pittsburgh Sleep Quality Index; REM, rapid eye movement sleep; SD, standard deviation.

### Tandem mass tag analysis of differentially expressed proteins

**FIGURE 2:**
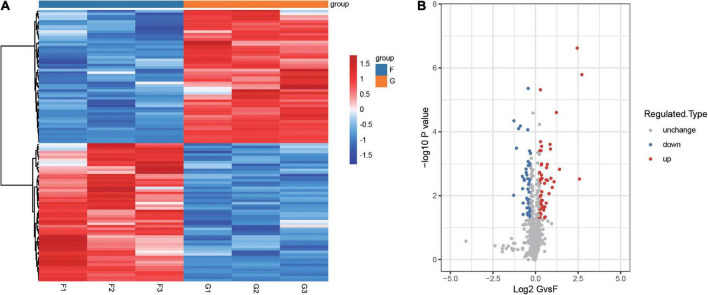
Identification of differentially expressed proteins between insomnia patients and controls. **(A)** Heatmap clustered with unsupervised for differentially expressed proteins between patients with insomnia and controls. F1-3 and G1-3 refer to technical replicates. **(B)** Volcano plot of differentially expressed proteins between patients with insomnia and controls. Red is upregulated proteins and blue is downregulated. G, insomnia group; F, control group.

We then performed TMT quantitative proteomics using five primary insomnia complaints and five controls (Table 2). To identify aberrantly expressed proteins in insomnia, we performed a differential analysis of expressed proteins between insomnia and control groups. First, 276,800 secondary spectra were obtained by TMT quantitative proteomics, and the number of available spectra was 16,913. A total of 5,218 peptide segments were identified by spectrogram analysis, in which the specific peptide segment was 5,020. We identified 836 proteins, 741 of which were quantifiable. A total of 106 DEPs were screened out of quantifiable proteins by the criteria described above (Figure 2A and Supplementary Table 1). We found that 52 proteins were upregulated and 54 proteins were downregulated in the insomnia group compared with controls (Figure 2B).

**TABLE 2:** Baseline characteristics of the participants in TMT quantitative proteomics.

Characteristic	Insomnia group	Healthy control group	*p*	*t*
Age, y				
Mean (SD)	40.6 (4.72)	43.6 (3.36)	0.283	1.16
BMI, mean (SD), kg/m^2^	20.53 (1.82)	25.64 (3.98)	0.03	2.61
Time in bed, min	534.8 (54.63)	523 (85.06)	0.80	0.26
Total sleep time, min	487.6 (40.12)	369.8 (87.15)	0.02	2.75
Sleep efficiency (%)	91.38 (3.96)	71.16 (15.07)	0.02	2.90
Sleep-onset latency, min	8 (8.95)	52.7 (20.76)	0.002	4.42
Awake time, min	37.5 (17.19)	82.8 (54.47)	0.11	1.77
Awakening times	5.4 (4.44)	11 (5.34)	0.11	1.80
REM sleep, min	83.4 (29.67)	74.6 (37.58)	0.69	0.41
REM%	17.08 (6.26)	19.38 (5.62)	0.56	0.61
N1%	12.1 (12.46)	18.52 (12.49)	0.44	0.81
N2%	61.48 (9.58)	57.24 (5.28)	0.41	0.87
N3%	9.32 (6.48)	4.82 (7.34)	0.33	1.03
NREM sleep, min	404.2 (43.61)	295.2 (54.79)	0.008	3.48
NREM%	82.92 (6.27)	80.61 (5.63)	0.56	0.61
Sex				
Male	1	3	0.19	1.67
Female	4	2		

BMI, body mass index; NREM, non-rapid eye movement sleep; REM, rapid eye movement sleep; SD, standard deviation.

### Biological functions of differentially expressed proteins

**FIGURE 3:**
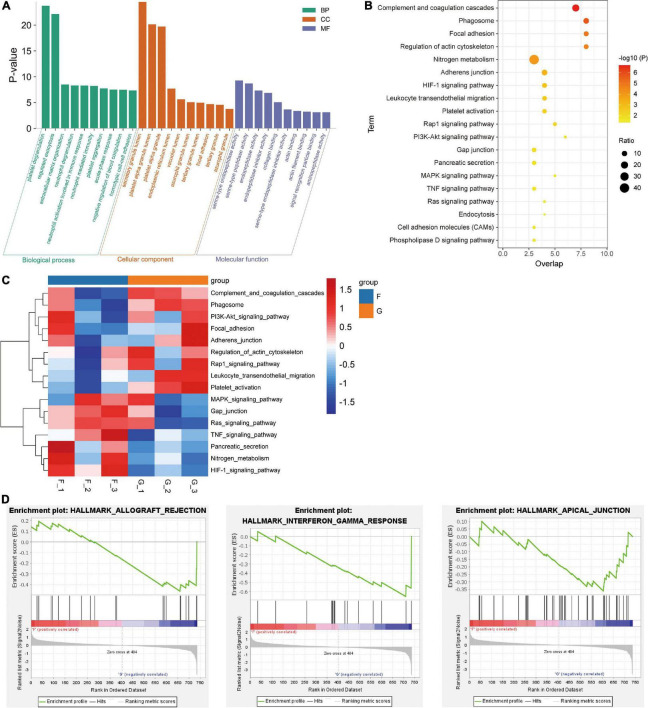
Identification of GO and signaling pathways enriched by differentially expressed proteins. **(A)** Top 10 terms of BP, CC, and MF enriched in insomnia patients. **(B)** KEGG pathways of DEPs significantly enriched. **(C)** Heatmap clustered with unsupervised for the activated or inhibited KEGG pathways were calculated by GSVA. **(D)** The significant hallmark terms positively corrected with patients with insomnia. G, insomnia group; F, control group.

To further explore the potential mechanism in patients with insomnia, we performed an enrichment analysis of DEPs. In the results of GO enrichment, the biological process (BP), cellular component (CC), and molecular function (MF) were included (Figure 3A). The DEPs are mainly located in platelet degranulation, neutrophil-mediated immunity, and extracellular matrix organization of BP. For the CC, DEPs were mainly enriched in secretory granule lumen, platelet alpha granule, and endoplasmic reticulum lumen. As for MF, DEPs were mainly involved in serine-type endopeptidase activity, endopeptidase activity, and collagen binding.

Meanwhile, 19 KEGG pathways were identified based on DEPs (Figure 3B), which mainly included complement and coagulation cascades, HIF-1 signaling pathway, and focal adhesion. We then identified the expression of these signaling pathways in different samples by GSVA (Figure 3C). Complement and coagulation cascades and focal adhesion were activated in patients with insomnia, and the HIF-1 signaling pathway was inhibited. In addition, GSEA was used to gain the hallmark (Figure 3D). We found that interferon-gamma response, allograft rejection, and apical junction were markedly enriched in the insomnia group.

### Protein–protein interaction analysis

**FIGURE 4:**
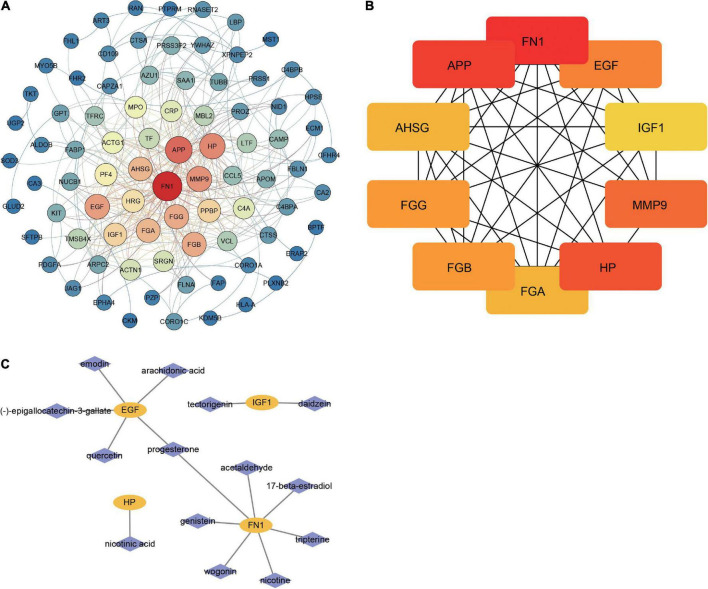
Identification of key proteins involved in insomnia. **(A)** The PPI network of DEPs. Nodes are colored from blue to red, indicating increased connectivity of proteins. **(B)** Top 10 proteins with the highest connectivity in the network. **(C)** Prediction network for natural compounds of herbal medicines targeting key DEPs.

STRING database was used to determine the relationship between DEPs. Of the 106 identified DEPs, 83 DEPs were interconnected (Figure 4A). The network exhibited a complex relationship among proteins. In addition, the Cytoscape software was served for PPI analysis of DEPs, the top 10 proteins with the largest connection degree (FN1, APP, HP, MMP9, EGF, FGG, FGB, AHSG, FGA, and IGF1) in the network were identified as key DEPs (Figure 4B). Key DEPs may have a huge impact on insomnia. Notably, we utilized the TCMSP database to identify potentially targeted drugs for key DEPs. In the results, we found FN1, EGF, HP, and IGF1 may be targeted by many natural compounds in herbal medicines (Figure 4C).

### Validation of differentially expressed proteins

**FIGURE 5:**
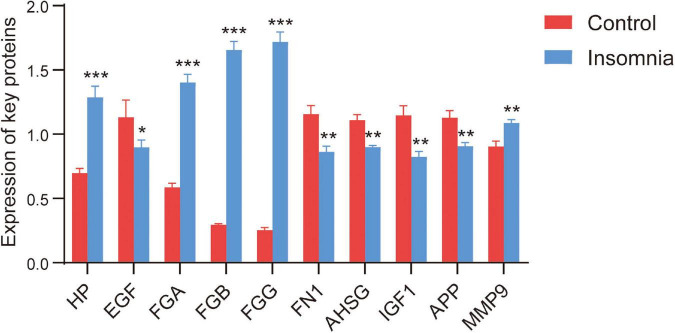
The expression of key DEPs. The expression of key DEPs in insomnia patients and controls in TMT-based proteomics. *p < 0.05, **p < 0.01, ***p < 0.001.

For TMT quantitative proteomics, we selected 15 patients with insomnia and 15 controls (Table 3). In the results, HP, FGA, FGG, FGB, and MMP9 were upregulated in patients with insomnia, and FN1, APP, EGF, AHSG, and IGF1 were downregulated compared with controls (Figure 5). Importantly, through PRM analysis for 106 DEPs, we quantified 34 DEPs (Table 4). Among them, the differential expression for eight key DEPs was validated. Among them, HP, MMP9, FGA, FGB, and FGG were validated as upregulated, and APP, AHSG, and IGF1 were downregulated in patients with insomnia.

**TABLE 3:** Baseline characteristics of the participants in PRM.

Characteristic	Insomnia group	Healthy control group	*p*	*t*
Age, y				
Mean (SD)	40.07 (8.00)	42.13 (5.05)	0.40	0.85
BMI, mean (SD), kg/m^2^	23.05 (2.22)	24.23 (3.73)	0.31	1.05
Time in bed, min	488.73 (24.12)	516.3 (73.9)	0.18	1.37
Total sleep time, min	452.2 (29.85)	395.47 (69.92)	0.007	2.89
Sleep efficiency (%)	92.5 (3.55)	76.83 (9.55)	<0.001	5.96
Sleep-onset latency, min	9.93 (12.05)	36.7 (19.59)	<0.001	4.51
REM sleep, min	56.8 (32.52)	63.8 (19.55)	0.48	0.71
REM%	12.51 (7.15)	16.31 (5.03)	0.10	1.69
N1%	12.89 (12.25)	9.08 (5.27)	0.28	1.11
N2%	65.39 (17.20)	70.26 (8.01)	0.32	0.99
N3%	9.2 (6.45)	4.35 (4.64)	0.03	2.36
Awakening times	3.13 (1.64)	11.6 (6.18)	<0.001	5.12
Awake time, min	25.17 (16.12)	64.67 (53.98)	0.01	2.72
NREM sleep, min	395.43 (39.03)	331.67 (66.99)	0.003	3.19
NREM%	87.50 (7.14)	83.68 (5.03)	0.10	1.69
Sex				
Male	5	4	0.69	0.159
Female	10	11		

BMI, body mass index; NREM, non-rapid eye movement sleep; REM, rapid eye movement sleep; SD, standard deviation.

**TABLE 4:** The results of PRM.

Protein accession	Protein gene	G relative abundance	F relative abundance	G/F ratio	G/F ratio (TMT)
P63261	ACTG1	1.05	0.95	1.10	1.28
**P02765**	AHSG	**0.87**	**1.13**	**0.76**	**0.81**
**P05067**	**APP**	**0.99**	**1.01**	**0.97**	**0.80**
O95445	APOM	0.82	1.18	0.69	1.24
P04003	C4BPA	1.22	0.78	1.57	1.27
P20851	C4BPB	1.18	0.82	1.44	1.35
Q6YHK3	CD109	1.40	0.60	2.34	0.83
P0C0L4	C4A	0.94	1.06	0.88	1.31
P36980	CFHR2	1.12	0.88	1.27	1.20
Q92496	CFHR4	1.34	0.66	2.03	1.25
P02741	CRP	1.29	0.71	1.83	1.43
P08294	SOD3	0.40	1.60	0.25	0.47
**P02671**	**FGA**	**1.99**	**0.01**	**350.62**	**2.39**
**P02675**	**FGB**	**1.99**	**0.01**	**248.67**	**5.58**
**P02679**	**FGG**	**1.99**	**0.01**	**354.88**	**6.74**
P02751	FN1	1.36	0.64	2.14	0.75
P23142	FBLN1	1.25	0.75	1.67	0.82
P21333	FLNA	1.22	0.78	1.56	1.25
P05062	ALDOB	1.58	0.42	3.72	2.03
**P00738**	**HP**	**1.40**	**0.60**	**2.36**	**1.84**
P26927	MST1	0.93	1.07	0.87	0.75
P04196	HRG	1.18	0.82	1.43	0.79
P01861	IGHG4	1.12	0.88	1.28	0.60
**P05019**	**IGF1**	**0.99**	**1.01**	**0.99**	**0.72**
P10619	CTSA	1.12	0.88	1.27	0.76
P11226	MBL2	0.68	1.32	0.51	0.42
**P14780**	**MMP9**	**1.46**	**0.54**	**2.68**	**1.20**
P36955	SERPINF1	1.14	0.86	1.32	0.83
P02775	PPBP	0.96	1.04	0.93	0.81
P02776	PF4	1.75	0.25	6.95	2.71
P20742	PZP	1.26	0.74	1.69	1.65
P10124	SRGN	1.06	0.94	1.14	0.75
P02787	TF	1.20	0.80	1.51	0.80
P22891	PROZ	0.97	1.03	0.94	0.72

G, insomnia group; F, control group. Values/terms were bolded for eight key DEPs.

## Discussion

Although evidence-based approaches to the assessment and treatment of insomnia have been developed, all are based on clinical consensus (Kalmbach et al., 2019). Further progress depends on a better understanding of the etiology and pathophysiology of mental health problems. Despite recent advances in the understanding of the nature, etiology, and pathophysiology of insomnia, there is no generally accepted model (Levenson et al., 2015). This study combined relevant biological information to identify important proteins in patients with insomnia and tried to reveal herbal medicines for targeted therapy.

The total sleep time of the patients with insomnia who participated in this study was short, while insomnia time and awakening times were significantly higher than those of the control group, which was consistent with the study of Johann et al. (2017). Interestingly, patients with insomnia had no significant difference between rapid eye movement sleep (REM) and controls, whereas non-rapid eye movement sleep (NREM) was significantly lower than controls. Most insomnia research has focused on quantitative electroencephalogram (EEGs) obtained during NREM sleep, rather than REM sleep, and differences in the control of insomnia have largely focused on EEG activity in the NREM during the night (Buysse et al., 2008).

Based on the proteomic quantification results, we identified a large number of DEPs. The enrichment results of DEPs identified biological functions and signaling pathways related to insomnia. It has been shown that insufficient sleep has a great impact on our immune system (Irwin and Opp, 2017). Christoffersson et al. (2014) found that a decreased sleep also decreased reactive oxygen species production by neutrophils. However, the existing studies on the relationship between insomnia and neutrophils are still less, and the specific effect mechanisms between the two need to be further investigated. Platelet degranulation is often associated with the innate immune response and may be involved in the regulation of physiological sleep (Besedovsky et al., 2019). Extracellular matrix (ECM) organization has been reported to be involved in the occurrence and development of insomnia (Xu et al., 2020). MMP9 (matrix metallopeptidase 9) is involved in the proteolytic modification of ECM proteins, which affects neuronal plasticity and the synaptic properties of neurons (Hayashi et al., 2013). In addition, melatonin has also a regulatory effect on the MMP9 pathway and ECM remodeling (Jensen et al., 2021). These results further confirmed a link between the immune-inflammatory response and insomnia.

In the KEGG pathway results, complement and coagulation cascades, and focal adhesion were activated by DEPs in patients with insomnia, and the HIF-1 signaling pathway was inhibited. Complement and coagulation play a role in the pathophysiology of REM sleep behavior disorder (Mondello et al., 2018). Complement and coagulation cascades were shown to stimulate the production of circadian regulatory hormones (Budkowska et al., 2018). Proteins are regulated by the coagulation cascade can disturb the homeostasis of synapses, affecting the pathophysiology of the central nervous system (CNS) (De Luca et al., 2017). Targeting the Ros-HIF-1 axis may enable the treatment of obstructive sleep apnea-related cardiovascular complications (Belaidi et al., 2016). In addition, the study showed that nurses with short sleep duration and poor sleep quality had elevated interferon gamma levels (Bjorvatn et al., 2020). Excessive secretion of interferon-gamma in the periphery and the brain triggers inflammatory cascades involved in aging and aging-related medical and psychiatric disorders (Oxenkrug, 2011).

Additively, this study also explored the interaction network between DEPs, and in turn screened out key DEPs (FN1, APP, HP, MMP9, EGF, FGG, FGB, AHSG, FGA, and IGF1) that may have important effects on insomnia. Importantly, upregulated HP, MMP9, FGA, FGB, FGG, and downregulated APP, AHSG, and IGF1 were validated by PRM analysis. HP (haptoglobin) is an inflammatory marker whose expression levels are significantly increased in patients with insomnia (Maes et al., 1993; Mominoki et al., 2005). MMP9 is an inflammatory protein that proved to be a potential target of action in the treatment of insomnia (Lee et al., 2012). High expression of fibrinogen is associated with more sleep problems (von Kanel et al., 2018). APP (amyloid-beta precursor protein) mutations severely affect sleep status in Alzheimer’s disease model mice (Huitron-Resendiz et al., 2005). Acute sleep deprivation elevates amyloid β-protein (Aβ) levels in mouse interstitial fluid and human cerebrospinal fluid (CSF) (Shokri-Kojori et al., 2018). In cardiovascular disease, the levels of AHSG (alpha 2-HS glycoprotein) are significantly lower than in controls (Zheng et al., 2014). However, since direct reports of its involvement in insomnia have not been found, it may be a new marker for insomnia. Reduced levels of IGF1 (insulin-like growth factor 1) were found to be associated with poor sleep quality, excessive daytime sleepiness, and sleep apnea (Reddy et al., 2014). Our results further emphasize abnormalities of these proteins in the process of insomnia.

The pharmacotherapy of insomnia has been changing over the past few decades, and traditional Chinese medicine (TCM) has been widely used for the treatment of insomnia due to its advantages, such as high efficacy, no drug resistance, and low toxicity (Singh and Zhao, 2017; Yue et al., 2019). For drug prediction of key DEPs, FN1, EGF, HP, and IGF1 were found to be potentially target-regulated by natural compounds. Therefore, they may be potential herbal therapeutic targets for patients with insomnia. Genistein contained in soja (*Glycine max*) and red clover (*Trifolium pratense* L.) was beneficial in antioxidant, anti-inflammatory, cardiovascular, and anxiolytic-like effects (Sharifi-Rad et al., 2021). Recent studies have shown that genistein can improve brain function through the blood–brain barrier, antagonize Aβ protein toxicity, and has neuroprotective effects (Duan et al., 2021). The 17-beta-estradiol is reported to moderately reduce insomnia symptoms and improve subjective sleep quality (Ensrud et al., 2015). Cohrs et al. (2014) found that sleep quality and sleep duration are impaired in nicotine-dependent inhalers. Acetaldehyde, a central nervous system depressant, has sedative, as well as sleep–wake cycle disturbances (Plescia et al., 2021). Wogonin inhibits inflammatory responses and, in turn, exerts neuroprotective effects (Piao et al., 2004). Arachidonic acid is a precursor for the production of prostaglandin PGD2, which is essential for sleep quality (Sejbuk et al., 2022). Emodin has various pharmacological properties, including anti-inflammatory, antioxidant, and neuroprotective effects (Li et al., 2021). Quercetin is one of the most potent antioxidants of plant origin, and its neuroprotective effects have been reported in several *in vitro* studies (Khan et al., 2019). This may provide new or improved therapeutic strategies in clinical practice.

This study also has some limitations. Limited by the number of experimental samples, not many DEPs were identified, although our filter condition was set at *p* < 0.05. The systemic findings reported here with CNS changes were important, while some of the altered serum proteins may reflect changes occurring in the brain, this is likely not the case for all proteins. In addition, the identified protein changes may be a cause or a consequence of insomnia; this need to be further explored in *in vivo* and *in vitro* experiments. In a follow-up study, we will focus on the expression changes of 10 key DEPs in patients with different degrees of insomnia in a large sample size.

## Conclusion

In this study, we applied TMT quantitative proteomic and bioinformatic analysis to explore possible mechanisms of patients with insomnia. After a series of detection, we discovered 106 DEPs associated with complement and coagulation cascades and HIF-1 signaling pathway. Then, we screened 10 key DEPs and validated them by PRM. This study has the potential to add to the current knowledge of pathophysiology of insomnia.

## Data availability statement

The datasets presented in this study can be found in online repositories. The name of the repository and accession number can be found below: ProteomeXchange Consortium, http://www.proteomexchange.org/, PXD026610.

## Ethics statement

Written informed consent was not obtained from the individual(s) for the publication of any potentially identifiable images or data included in this article.

## Author contributions

TL designed this work, collected samples, and drafted the manuscript. GW and XL performed data analysis and pathway analysis. ZL and XR interpreted data and conducted experiments. DY and WZ performed the statistical analyses. All authors contributed to manuscript development and had approved the final and submitted draft of the manuscript.

## Conflict of interest

The authors declare that the research was conducted in the absence of any commercial or financial relationships that could be construed as a potential conflict of interest.

## Publisher’s note

All claims expressed in this article are solely those of the authors and do not necessarily represent those of their affiliated organizations, or those of the publisher, the editors and the reviewers. Any product that may be evaluated in this article, or claim that may be made by its manufacturer, is not guaranteed or endorsed by the publisher.

## Footnotes

1
http://www.string-db.org/

## Supplementary material

Supplementary Table 1 The differentially expressed proteins between patients with insomnia and controls.
